# Efficient Synthesis of Peptide and Protein Functionalized Pyrrole-Imidazole Polyamides Using Native Chemical Ligation

**DOI:** 10.3390/ijms160612631

**Published:** 2015-06-04

**Authors:** Brian M. G. Janssen, Sven P. F. I. van Ommeren, Maarten Merkx

**Affiliations:** Laboratory of Chemical Biology and Institute for Complex Molecular Systems, Eindhoven University of Technology, Den Dolech 2, 5600 MB Eindhoven, The Netherlands; E-Mails: b.m.g.janssen@tue.nl (B.M.G.J.); svenvanommeren@gmail.com (S.P.F.I.V.O.)

**Keywords:** pyrrole-imidazole polyamides, DNA-functionalization, native chemical ligation, DNA-nanotechnology, surface plasmon resonance

## Abstract

The advancement of DNA-based bionanotechnology requires efficient strategies to functionalize DNA nanostructures in a specific manner with other biomolecules, most importantly peptides and proteins. Common DNA-functionalization methods rely on laborious and covalent conjugation between DNA and proteins or peptides. Pyrrole-imidazole (Py–Im) polyamides, based on natural minor groove DNA-binding small molecules, can bind to DNA in a sequence specific fashion. In this study, we explore the use of Py–Im polyamides for addressing proteins and peptides to DNA in a sequence specific and non-covalent manner. A generic synthetic approach based on native chemical ligation was established that allows efficient conjugation of both peptides and recombinant proteins to Py–Im polyamides. The effect of Py–Im polyamide conjugation on DNA binding was investigated by Surface Plasmon Resonance (SPR). Although the synthesis of different protein-Py–Im-polyamide conjugates was successful, attenuation of DNA affinity was observed, in particular for the protein-Py–Im-polyamide conjugates. The practical use of protein-Py–Im-polyamide conjugates for addressing DNA structures in an orthogonal but non-covalent manner, therefore, remains to be established.

## 1. Introduction

In addition to its natural role in storing genetic information, DNA has become a versatile building block for a wide range of applications in bionanotechnology [1]. The synthetic accessibility and highly predictable binding properties of DNA allow the bottom up construction of complex 3-dimensional DNA nanostructures with well-defined geometries, topologies and mechanical properties [2,3]. The development of efficient strategies to address DNA nanostructures with functional proteins and peptides is important for their use in biomedical applications [4,5,6]. The most common strategy to spatially address DNA-nanoarchitectures with proteins is via covalent and chemoselective conjugation (e.g., thiol-maleimide, “click”-chemistry) of the protein with ssDNA, which subsequently hybridizes to complementary handles present on the DNA nanostructure [5,7]. These approaches require the introduction of single-strand DNA handles during the design of the DNA architectures and inevitably introduces a rigid dsDNA linker of >5 nm between the protein and the DNA-architectures. Another popular strategy is to use biotinylated-oligonucleotides and the very strong streptavidin-biotin interaction to immobilize biotinylated proteins or peptides [8,9]. However, in addition to the introduction of the relatively large streptavidin linker, this strategy often yields heterogeneous complexes due to the tetravalent nature of streptavidin and the essentially irreversible nature of the interaction. Self-assembly of protein-DNA assemblies has also been achieved by introduction of DNA aptamer sequences, but this strategy is restricted to the limited number of proteins for which high affinity aptamers have been developed such as thrombin [10,11].

Instead of the introduction of specific protein binding oligonucleotide tags, protein functionalization of DNA-based nanostructures can also be achieved using sequence-specific dsDNA binders such as zinc finger domains or Py–Im polyamides. Recent examples of zinc fingers include the functionalization of DNA origami structures with fluorescent proteins [12] and the re-assembly of a split luciferase for DNA detection [13] and logic-gated protein function [14]. Although zinc finger domains can be connected to the protein of interest by genetic fusion, functional expression of the cysteine-rich zinc binding proteins can be challenging. Py–Im hairpin polyamides were developed by Dervan and coworkers as a low molecular weight alternative to DNA binding proteins [15,16]. Inspired by the natural products netropsin and distamycin, these polyamide oligomers of pyrrole, imidazole and hydroxypyrrole can be designed to bind in a sequence specific manner in the minor groove of dsDNA with nanomolar affinity. Paired imidazole (Im) and pyrrole (Py) recognize G–C basepairs whereas paired Py/Im recognize C–G. Py/Py recognizes both A–T and T–A basepairs [17,18,19]. Research on Py–Im polyamides initially focused on the control of gene transcription by targeting transcription factor recognition sites [16,20,21,22]. Activation of gene transcription was achieved by synthesizing peptide-Py–Im-polyamide conjugates of which the peptide was able to recruit transcription factors and thereby initiate gene transcription at the site of interest [23,24,25,26]. More recently Py–Im polyamides have been used as specific dsDNA binders in other applications, including DNA-detection based on the aggregation of gold nanoparticles [27,28] or microcontact printing [29], as a construction element for DNA architectures [30,31] or controlled energy transfer along a DNA wire [32]. The use of Py–Im polyamides for the sequence specific patterning of streptavidin on a DNA-scaffold has been achieved by attaching a biotin group via a polyethylene glycol (PEG) linker to Py–Im polyamides [33,34,35].

Py–Im polyamide conjugates are typically obtained by using classical amine coupling reactions, yielding Py–Im polyamide conjugates containing (protected) peptides [24,26] or small molecules like fluorophores [36,37]. Native chemical ligation has been explored to accelerate the reaction between distamycin-related polyamides equipped with cysteine and thioester groups in the presence of a cognate DNA sequence [38] and also as a more chemoselective reaction to couple non-protected peptides to Py–Im polyamides [23,39]. Mapp and coworkers treated the C-terminus of the Py–Im polyamide with benzyl bromide to produce a thioester and subsequently conjugated the Py–Im polyamide to an N-terminal cysteine present on a glutamate-rich peptide to facilitate the recruitment of transcription factors to specific DNA sites [23,39].

**Scheme 1:**
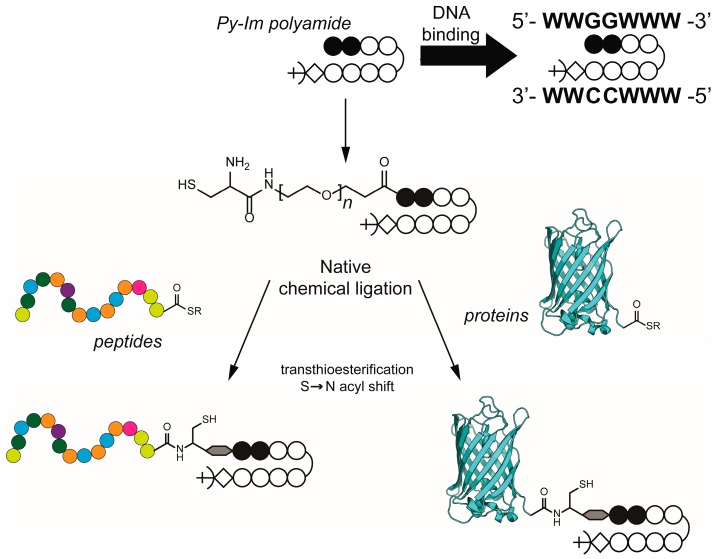
Synthesis of pyrrole-imidazole (Py–Im) polyamide conjugates via native chemical ligation. Schematic representation of the Py–Im polyamide sequence used in this study (ImImPyPy-γ-PyPyPyPy-β-Dp) to target the DNA sequence 5ʹ-WWGGWWW-3ʹ (W represents either A or T). The introduction of a cysteine at the N-terminus of the Py–Im polyamide allows native chemical ligation of peptides and proteins bearing a C-terminal thioester.

The aim of this study was to develop a generic synthetic strategy for the conjugation of Py–Im polyamides to peptides and recombinant proteins. Unlike the previous reported strategy of introducing a thioester on the Py–Im polyamide, our strategy was to functionalize Py–Im polyamides with an N-terminal cysteine. Introduction of the more stable cysteine on the Py–Im polyamide is advantageous because synthesis of the Py–Im polyamide is the most labor intensive step (Scheme 1). These cysteine-functionalized Py–Im polyamides were shown to readily react with peptides and proteins containing a C-terminal thioester. Characterization of the DNA binding properties of these Py–Im polyamide conjugates using Surface Plasmon Resonance revealed a significant attenuation of DNA affinity, in particular for the protein-Py–Im-polyamide conjugates.

## 2. Results

### 2.1. Synthesis of Cysteine-Functionalized Pyrrole-Imidazole (Py–Im) Polyamide

**Scheme 2:**
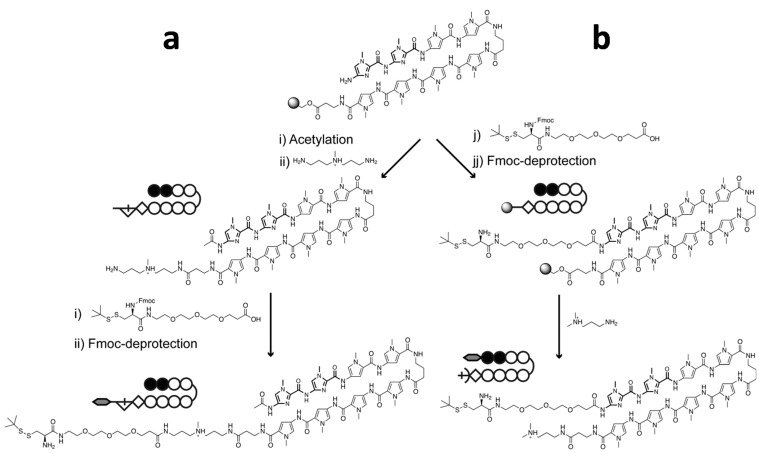
Synthetic scheme for the preparation of Py–Im polyamide bearing a PEGylated and *tert*-butylthiol protected cysteine for native chemical ligation purposes. The *tert*-butylthiol protected cysteine can be introduced at the C-terminus (**a**) or N-terminus (**b**) of the Py–Im polyamide. Introduction at the C-terminus requires cleavage from the resin using *N*,*N*-bis(aminopropyl)methylamine (BDp) followed by conjugation of the pre-synthesized PEG_3_-Cys(StBu) to the free amine of BDp. Introduction of PEGylated and *tert*-butylthiol protected cystein at the N-terminus can be performed on resin followed by cleavage from the resin using 3-(dimethylamino)-propylamine (Dp).

In this work, we used ImImPyPy-γ-PyPyPyPy-Dp as a model Py–Im polyamide. This sequence was chosen because it avoids the use of the hydrolytically sensitive hydroxypyrrole [40], but is still reasonably specific because of the use of two consecutive imidazoles. Furthermore, this Py–Im polyamide was reported to bind dsDNA 5ʹ-WWGGWWW-3ʹ (W represents either A or T) with 2 nM affinity [41]. Native chemical ligation of peptides and proteins with a C-terminal thioester requires the introduction of a cysteine with a free N-terminal amine in the Py–Im polyamide. This cysteine could be introduced at either terminus of the Py–Im polyamide (Scheme 2).

Introduction at the C-terminal part of the Py–Im polyamide requires cleavage from the resin by *N*,*N*-bis(aminopropyl)methylamine (BDp), followed by a coupling step in solution to the pre-synthesized PEGylated and *tert*-butylthiol protected cysteine. Although the final product was obtained in pure form, synthesis of the C-terminal cysteine-modified Py–Im polyamide was rather cumbersome due to the final coupling in solution, resulting in low overall yield of 3% after HPLC-purification. A synthetically more straightforward approach is to introduce the cysteine at the N-terminus of the Py–Im polyamide, since in this case all coupling steps can be done on the resin. First, we synthesized the polyamide ImImPyPy-γ-PyPyPyPy manually using Fmoc-β-alanine-Wang resin [40]. To prevent steric hindrance between the protein and the DNA, we chose to first introduce a short PEG_3_-linker at the N-terminus. Following deprotection of the Fmoc-group, the amine of the PEG_3_-linker was coupled to Fmoc- and *t*-BuSH-protected cysteine on the solid phase, followed by Fmoc-deprotection and cleavage from the solid-phase.

**Figure 1:**
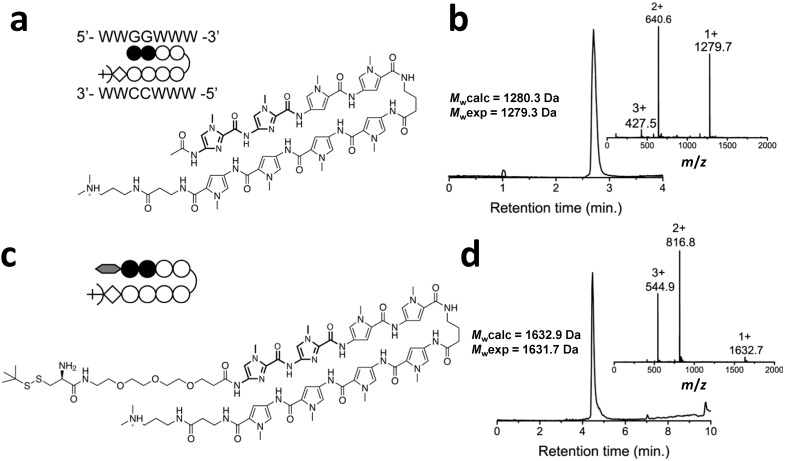
(**a**) Chemical structure and schematic representation of the reference Py–Im polyamide; (**b**) RP-HPLC and *m*/*z*-spectra of the purified acetylated Py–Im polyamide; (**c**) Chemical structure and schematic representation of the Py–Im polyamide sequence containing the PEG_3_-linker and *t*-BuSH-protected cysteine (Cys-Py–Im-polyamide) after cleavage from the resin using Dp; (**d**) RP-HPLC and *m*/*z*-spectra of the purified Cys-Py–Im-polyamide. *M*_w_exp was obtained after deconvolution of the *m*/*z*-spectrum. *M*_w_calc is the calculated average molecular weight.

Figure 1 shows the chemical structure and electro spray ionization mass spectrometry (ESI-MS) characterization of the final product, Cys-Py–Im-polyamide, as well as the Py–Im polyamide reference compound with an N-terminal acetyl group. Both compounds were cleaved via aminolysis of the ester on the resin using 3-(dimethylamino)-propylamine (Dp). The positively charged tertiary amine that is formed at the C-termini of the Py–Im polyamides provides an additional electrostatic interaction between the Py–Im polyamide and DNA. Typical overall yields after reversed phase high-performance liquid chromatography (RP-HPLC) were 20% for the Py–Im polyamide reference and 14% for Cys-Py–Im-polyamide, which is comparable to yields reported previously for Py–Im polyamides of similar sequences [40].

### 2.2. Native Chemical Ligation of Thioester Peptides and Proteins to Cys-Py–Im-Polyamides

**Figure 2:**
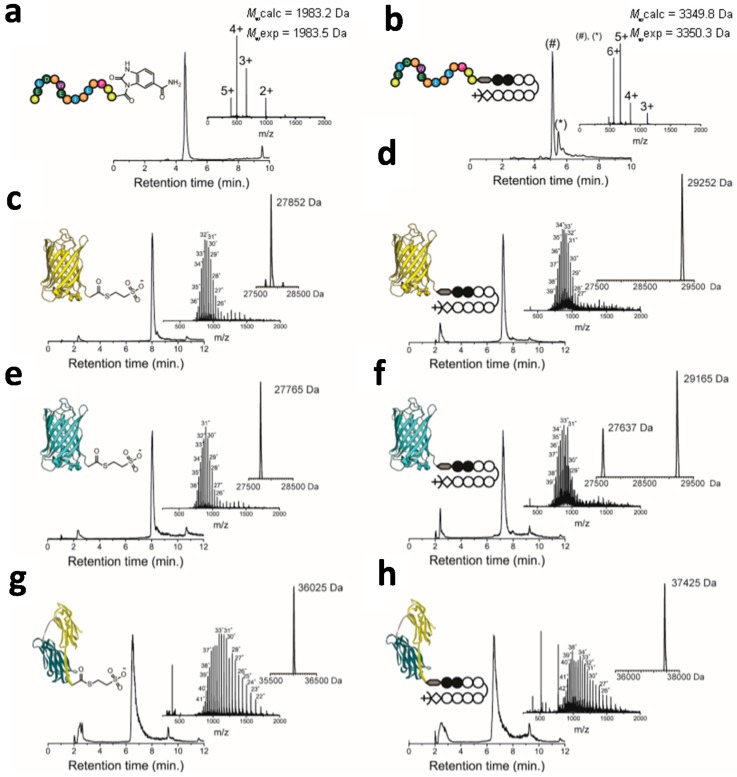
Native chemical ligation of peptides and proteins with a C-terminal thioester and Cys-Py–Im-polyamide. (**a**) RP-HPLC and *m*/*z*-spectrum of the purified *N*-acyl-benzimidazolinone (Nbz) anti-HIV epitope peptide; (**b**) RP-HPLC and *m*/*z*-spectrum of the purified NCL-reaction between Cys-Py–Im-polyamide and Nbz-aHIV-epitope in 200 mM Na_2_HPO_4_, 200 mM MPAA, 20 mM TCEP·HCl, 6 mM Guanidine·HCl at pH 6.8. The # and * indicate different diastereomers with the same ESI-MS spectrum; (**c**,**e**,**g**) RP-HPLC and *m*/*z*-spectra of the purified EYFP-MESNA (*M*_w_calc = 27,845 Da, *M*_w_exp = 27,852 Da) (**c**), ECFP-MESNA (*M*_w_calc = 27,763 Da, *M*_w_exp = 27,765 Da) (**e**) and CNA35-MESNA (*M*_w_calc = 36,028 Da, *M*_w_exp = 36,025 Da) (**g**); (**d**,**f**,**h**) RP-HPLC and *m*/*z*-spectra of protein-Py–Im-polyamide conjugates of EYFP (*M*_w_calc = 29,254 Da, *M*_w_exp = 29,252 Da) (**d**), ECFP (*M*_w_calc = 29,167 Da, *M*_w_exp = 29,165 Da) (**f**) and CNA35 (*M*_w_calc = 37,427 Da, *M*_w_exp = 37,425 Da) (**h**); The additional peak at 27,637 Da corresponds to ECFP (**f**) in which the MESNA-thioester was hydrolysed to a carboxylic acid. All reactions with recombinant proteins were performed at room temperature in 200 mM Na_2_HPO_4_, 100 mM MPAA, 20 mM TCEP·HCl at pH 6.8. *M*_w_exp was obtained after deconvolution of the *m*/*z*-spectrum. *M*_w_calc is the calculated average molecular weight.

To test the efficiency of the native chemical ligation (NCL) to Cys-Py–Im-polyamide, a C-terminal thioester functionalized peptide epitope (ELDRWEKIRLRP) was synthesized that specifically binds to an anti-HIV p17 antibody [42,43]. The C-terminal thioester peptide was synthesized according to the method of Dawson and coworkers and involved Fmoc-solid phase synthesis using a Rink amide aminomethyl (AM) resin containing a diaminobenzyl linker [44]. The resin-bound *N*-acyl-benzimidazolinone (Nbz) peptide was deprotected and cleaved from the resin using trifluoroacetic acid. The desired peptide AELDRWEKIRLRPAA-Nbz was obtained in high purity and a final yield of 15% after RP-HPLC purification (Figure 2a). Since the Nbz-moiety is a poor leaving group, the NCL-reaction with Cys-Py–Im-polyamide was performed using 4-mercaptophenylacetic acid (MPAA) as a catalyst [45]. Cys-Py–Im-polyamide was mixed with a slight excess of peptide-Nbz to yield final concentrations of 2 and 2.2 mM, respectively. Analysis by ESI-MS showed full conversion of the Cys-Py–Im-polyamide to the epitope-Py–Im-polyamide conjugate after overnight incubation at room temperature (Figure 2b). The HPLC-trace actually showed two peaks with the expected mass for the epitope-Py–Im-polyamide product, which we attribute to the formation of a small amount of a diastereomer.

NCL-reactions with full-sized proteins tend to be less efficient compared to those involving peptide thioesters, if only because of the lower concentrations that can be used. Following successful NCL with a peptide thioester, we therefore next tested the efficiency of the reaction between Cys-Py–Im-polyamide and three different recombinant proteins with a C-terminal MESNA-thioester. Enhanced yellow fluorescent protein (EYFP) and cyan fluorescent protein (ECFP) were chosen as they form a useful Förster resonance energy transfer (FRET) pair to allow assessment of protein-protein distances on DNA structures [46,47,48]. The collagen binding protein CNA35 has been successfully applied as a recognition domain for collagen imaging and was successfully used to construct CNA35-micelles [49], liposomes [50], and dendrimers [51]. C-terminal fusion proteins with intein and chitin binding domains were expressed in *E. coli* and purified using the intein mediated purification affinity chitin tag (IMPACT) method [50]. ESI-MS analysis showed the successful formation of 4-mercaptophenylacetic acid (MESNA) functionalized proteins (Figure 2c,e,g). The NCL-reaction was performed by mixing a 10-fold excess of a Cys-Py–Im-polyamide (3.0 mM) to protein-MESNA (0.3 mM). ESI-MS analysis showed full conversion to the ligation product after 36 h for all three proteins (Figure 2d,f,h). Excess of Cys-Py–Im-polyamide, TCEP and MPAA was extensively removed by exchanging buffer using centrifugal filters. Interestingly, conjugation of the Cys-Py–Im-polyamide did not affect the fluorescence of the fluorescent proteins, which is in contrast to previous reports for organic dyes where conjugation resulted in a substantial attenuation of fluorescence intensity (Figure S1). Whereas organic fluorophores may interact with the imidazole and pyrrole groups, the protein’s fluorophore is in the interior of the protein and protected by its β-barrel structure.

### 2.3. Effect of Conjugation on DNA Binding Properties

**Figure 3:**
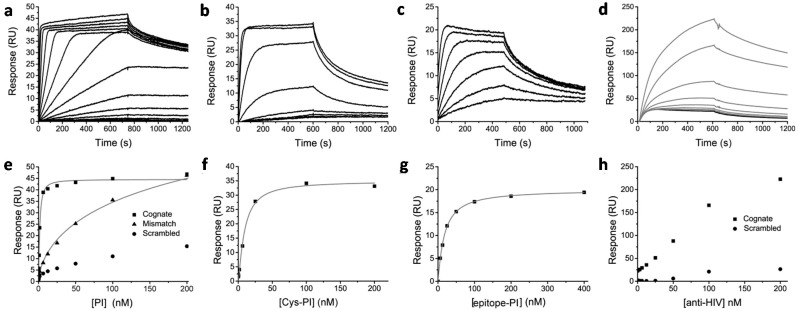
Surface plasmon resonance (SPR) titration experiments to study the effect of conjugation on DNA binding properties. Binding of non-functionalized Py–Im polyamide (**a** + **e**), Cys-Py–Im-polyamide (**b** + **f**), epitope-Py–Im-Polyamide conjugate (**c** + **g**) to the cognate DNA sequence during 8 to 12 min injections (25 µL·min^−1^) over a concentration range of 24 pM to 400 nM in HBS-EP + 0.1% DMSO, pH 7.4; (**e**) The steady-state binding levels of non-functionalized Py–Im polyamide were fitted individually to a one-site binding model yielding an overall *K*_d_ of 1.6 ± 0.1 nM for the non-functionalized Py–Im polyamide binding to the cognate DNA sequence and a *K*_d_ of 314 ± 160 nM for the 3 bp mismatch sequence; (**f**,**g**) The steady–state binding levels of Cys-Py–Im-polyamide and the epitope-Py–Im polyamide conjugate to the cognate DNA sequence resulted in an overall *K*_d_ of 9.3 ± 1.6 and 17.4 ± 0.5 nM, respectively; (**d**) Response of pre-incubated mixtures containing 100 nM epitope-Py–Im-polyamide conjugate (black line) and anti-HIV antibody binding in a concentration range of 0 to 200 nM (grey lines) to the cognate DNA sequence; (**h**) Binding level as a function of antibody concentration for the cognate and scrambled DNA sequences.

Previous work showed that peptide conjugation can affect both the affinity and specificity of Py–Im polyamides binding to DNA [23,24,25,52]. To study the influence of peptide and protein conjugation, the DNA binding properties of the Py–Im polyamide conjugates were investigated using surface plasmon resonance (SPR). Commercially available streptavidin-functionalized SPR chips were immobilized with biotin-functionalized oligonucleotides (biotin-ODN) that form a hairpin loop resulting in a piece of double stranded DNA containing the cognate sequence for the Py–Im polyamide of interest [53]. Besides the cognate DNA-sequence, we chose to immobilize a scrambled DNA sequence and a sequence containing three mismatches. Increasing concentrations of the reference Py–Im polyamide were injected and flown over the different channels containing one of the three DNA-sequences. The sensorgram of the matching DNA-sequence showed a low dissociation rate indicating tight binding of the non-functionalized Py–Im polyamide to the DNA. The maximum response level of approximately 40 RUs confirmed that the Py–Im polyamide molecule binds to the DNA in a 1:1 stoichiometry (Figure 3a). Fitting the steady state response levels with a 1:1 binding Hill equation (*n* = 1, no cooperativity) yielded a *K*_d_ of 1.6 ± 0.1 nM for the cognate DNA-sequence, which is comparable to the *K*_d_ of 2.0 nM reported previously for this Py–Im polyamide sequence using quantititative footprint titrations [41]. In accordance with previous work, a ~100-fold decreased affinity was observed for a sequence that contained three mismatches compared to the cognate DNA sequence (Figure 3e) [54]. Interestingly, introduction of the cysteine-functionalized PEG_3_-linker at the N-terminus of the Py–Im polyamide resulted in a sixfold decrease in affinity towards the cognate DNA sequence (Figure 3b,f), yielding an apparent *K*_d_ of 9.3 ± 1.6 nM. The decreased affinity observed for Cys-Py–Im-polyamide was at least partially due to an enhanced dissociation rate (Figure 3b).

A further twofold decrease in DNA affinity to a *K*_d_ of 17.4 ± 0.5 nM was observed for the epitope-Py–Im-polyamide conjugate (Figure 3c,e). This relatively small additional effect of peptide conjugation suggests that most of the attenuation in DNA affinity is due to local steric and/or repulsive effects also present in Cys-Py–Im-polyamide. To test whether the peptide epitope is still available for antibody binding when the epitope-Py–Im-polyamide is bound in the DNA minor groove, a fixed concentration of 100 nM epitope-Py–Im-polyamide was pre-incubated with different anti-HIV antibody concentrations ranging from 0 to 200 nM. Figure 3d shows that there is an antibody concentration dependent increase in response. Although complex formation is not complete under these conditions, this experiment shows that the epitope-Py–Im-polyamide conjugate enables the functionalization of DNA with, in this case, antibodies in a non-covalent and a sequence specific manner (Figure 3h).

**Figure 4:**
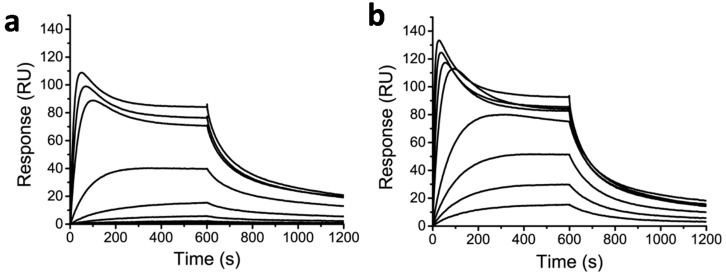
Binding of protein-Py–Im-polyamide conjugate to a surface modified with the cognate DNA sequence. (**a**) Response of ECFP-Py–Im-polyamide conjugate binding in a concentration range of 1.56 to 400 nM to the cognate DNA sequence; (**b**) Response of CNA35-Py–Im-polyamide conjugate binding in a concentration range of 1.56 to 400 nM binding to the cognate DNA sequence. Experiments were conducted in HBS-EP + 0.1% DMSO, pH 7.4 and using 10 min injections (25 µL·min^−1^).

Finally, the SPR binding assay was used to assess the DNA-binding properties of the protein-Py–Im-polyamide conjugates (Figure 4). Although DNA binding was clearly observed, both the ECFP-Py–Im-polyamide and the CNA35-Py–Im-polyamide conjugate showed complex association kinetics. At relatively high concentrations, the initial rapid increase in response was followed by an unexpected drop in response levels until a steady-state level was reached. A possible explanation for this complex binding behavior could be competitive binding by a compound with a higher affinity and lower molecular weight. Care was taken to remove the excess of non-conjugated Cys-Py–Im-polyamide following NCL, either be repeated concentration/dilution cycles using centrifugal filters (for ECFP and EYFP) or using Ni-affinity chromatography (for CNA35). Nonetheless, we cannot exclude the possibility that some of the Cys-Py–Im-polyamide sticks to the protein due to its strongly hydrophobic nature. Although the steady-state levels are higher for the protein-Py–Im-polyamide conjugates compared to the smaller Cys-Py–Im-polyamide or epitope-Py–Im-polyamide conjugate, it is also clear that the binding is not saturated even at the highest concentration of protein-Py–Im-polyamide tested (400 nM), which indicates that the DNA affinity is further attenuated for the protein-Py–Im-polyamide conjugates compared to the Cys-Py–Im and epitope-Py–Im-polyamide conjugates.

## 3. Discussion and Conclusion

A novel synthetic strategy was developed that allows native chemical ligation of both peptides and proteins to Py–Im polyamides. The introduction of an N-terminal and thiol-protected cysteine on the Py–Im polyamide combined with established strategies to synthesize thioester-peptides and express thioester proteins resulted in quantitative conversion to the conjugation product [24,26], while avoiding the cumbersome introduction of a C-terminal thioester on the Py–Im polyamide upon treatment with benzyl bromide [23,39]. Our synthetic approach also compares favorably with previous work that used standard peptide coupling conditions to conjugate short side-chain protected peptide fragments (5-mers) to a free amine on the Py–Im-polyamide. Although the conjugation was successful, this strategy showed lower conversion (maximum of 60%) and required the use of excess peptide and an additional cleavage step after conjugation to obtain unprotected peptide-Py–Im-polyamide.

Our finding that the DNA binding of the epitope-Py–Im-polyamide was tenfold weaker compared to the reference Py–Im-polyamide is consistent with previous work on Py–Im polyamide conjugation [23]. Both the chemical nature and the length of the linker used for the peptide-Py–Im-polyamide conjugates are known to be important for maintaining the DNA-binding properties of the Py–Im-polyamide. A 100-fold affinity loss was observed upon conjugation of a gene-expression promoting peptide sequence to the *N*-methyl position of imidazole of a Py–Im polyamide via a linker consisting of a single glycine [24,26]. In a follow up study, the same peptide was connected at the C-terminus of the Py–Im polyamide via a PEG_3_-linker or equivalent alkyl linker containing a tertiary amine. The peptide-Py–Im-polyamide containing the tertiary amine was suggested to bind stronger to the DNA, since the recruitment of the transcription factor protein was more efficient compared to the conjugate containing the PEG-linker. The introduction of a PEG_4_-linker between a small molecule (camptothecin) and a Py–Im polyamide sequence resulted in a 10-fold loss in affinity compared to the non-functionalized, whereas an equivalent alkyl-linker resulted in 25-fold loss in affinity [52].

The further decrease in DNA affinity observed upon conjugation of full sized proteins to Cys-Py–Im-polyamide may by partially due to the ineffective removal of excess hydrophobic Cys-Py–Im-polyamide. In addition, the current PEG_3_-linker might be insufficient to separate the protein from the Py–Im polyamide, thereby hampering the binding of the latter to the DNA. To increase the spatial separation between the protein and the Py–Im polyamide one could introduce longer PEG-linkers or linkers with an increased persistence length. Another strategy would be to connect the protein to a tandem-Py–Im-polyamide. The affinity of these tandem-Py–Im-polyamides has been reported to be in the picomolar-range, but their synthesis is more laborious [55]. Finally, the Cys-PEG Py–Im polyamide could be functionalized with a chlorambucil-moiety, similar to previous reports [35]. Incubation of this Py–Im polyamide would result in a covalent bond with the DNA sequence of interest. In a subsequent step, the N-terminal cysteine on the Py–Im polyamide-DNA construct could react with thioester proteins (or peptides) to obtain protein-functionalized DNA structures. This approach might circumvent the attenuated affinity and dissociation of the non-covalent protein-Py–Im-polyamide conjugate from the DNA.

## 4. Experimental Section

See supplementary information for figure showing effect of Py-Im-polyamide conjugation on fluorescence of ECFP.

### 4.1. Fmoc-Mediated Solid Phase Synthesis of the Non- and Tert-BuSH Protected Cysteine Py–Im Polyamide

The synthesis of the Py–Im polyamide was performed manually on Fmoc-β-alanine-Wang resin (200 µmol scale, Novabiochem, Amsterdam, The Netherlands) following previously reported procedures [40].

N-terminal elongation: Typical cleavage of the synthesized Py–Im polyamide was achieved by the addition of 500 µL of 3-(dimethylamino)-propylamine (Dp, Sigma Aldrich, Zwijndrecht, The Netherlands) to the dried resin followed by 15 h of incubation at 37 °C under continuous agitation. Precipitation in diethyl ether was performed twice to obtain a white precipitate. For the Py–Im polyamide containing a cysteine at the N-terminus, Fmoc-12-amino-4,7,10-trioxadodecanoicacid (PEG_3_, PolyPeptide Group, Limhamn, Sweden) was coupled on resin followed by the coupling of Fmoc-*S*-*tert*-butylthio-l-cysteine (*t*-BuSH) (Novabiochem). No capping step was performed after deprotection of the Fmoc-Cys (*t*-BuSH). The resin was dried under vacuum and cleaved with Dp as described previously. Purification of the non-functionalized and the cysteine-functionalized Py–Im polyamide was performed by preparative RP-HPLC using a gradient of acetonitrile in H_2_O (both containing 0.1% TFA).

C-terminal elongation: The Py–Im polyamide was cleaved from the resin with *N*,*N*-bis(aminopropyl)methylamine (BDp) to obtain an amine functionality at the C-terminus of the Py–Im polyamide. 500 µL BDp was added to the dried resin followed by 15 h of incubation at 37 °C under continuous agitation. Precipitation was performed as described before. Fmoc-12-amino-4,7,10-trioxadodecanoicacid (PEG_3_) was coupled on 2-Chlorotrityl resin (100 µmol, Iris Biotech, Marktredwitz, Germany) according to standard peptide-coupling procedures. Fmoc-deprotection was performed followed by the coupling of Boc-protected *S*-*tert*-butylthio-l-cysteine (*t*-BuSH, Sigma Aldrich). A mixture of TFE/DCM (2/8 *v*/*v*) was added to the 2-Chlorotrityl resin and agitated for 3 h followed by the removal of DCM (and as much TFE as possible) *in vacuo* and the Boc-protected Cys(*t*-BuSH)-PEG with a C-terminal carboxylic acid was subsequently precipitated in a mixture of ice cold diethyl ether and hexane (2/1 *v*/*v*, respectively). After a centrifugation step, the pellet was dissolved in a mixture of acetonitrile and water, frozen and lyophilized. According to LC-MS analysis, one major peak was observed corresponding to Boc-Cys(*t*-BuSH)-PEG. A fivefold excess of Boc-Cys(*t*-BuSH)-PEG and HCTU were mixed with Py–Im polyamide bearing the C-terminal amine. The reaction in solution was performed in DMF and stirred overnight at room temperature. DMF was removed *in vacuo* followed by an additional cleavage step using a mixture of TFA/triisopropylsilane/water (95/2.5/2.5 *v*/*v*/*v*) to remove the Boc-group of the Cysteine. Finally, the Py–Im polyamide bearing the Cys(*t*-BuSH)-PEG at the C-terminus was precipitated in ice cold ether, lyophilized and purified with preparative RP-HPLC using a gradient of acetonitrile in H_2_O (both containing 0.1% TFA) resulting in a final yield of 3%.

### 4.2. Fmoc-Mediated Solid Phase Peptide Synthesis of Nbz-Peptide

The synthesis of the thioester peptide was performed manually on a Dawson Dbz AM resin (Novabiochem) on a 100 µmol scale following previously reported procedures [44]. After completion of the synthesis, the peptide was washed with dichloromethane (DCM) prior to the cleavage. 100 mg of *p*-nitrophenylchloroformate (Sigma-Aldrich) was dissolved in 6 mL DCM, added to the resin and agitated gently for one hour. The peptide was washed three times with DCM and twice with NMP followed by the addition of 0.5 M DIPEA and agitated for 30 min. After activation the peptide was washed with NMP and DCM. A mixture of TFA/triisopropylsilane/water (95/2.5/2.5 *v*/*v*/*v*) was added to the resin and agitated for 3 h followed by precipitation of the Nbz-peptide in ice cold diethyl ether. After a centrifugation step the pellet was dissolved in a mixture of acetonitrile and water, frozen, and lyophilized. Purification was done by preparative RP-HPLC using a gradient of acetonitrile in H_2_O (both containing 0.1% TFA). Gradient RP-HPLC: 18%–28% in 10 min with a final yield of 17%.

### 4.3. Protein Expression and Purification

The expression plasmids pTXB1-EYFP, pTXB1-ECFP and pTXB1-CNA35 were transformed in *E. coli* BL21 (DE3) cells. Similar expression conditions were used for both proteins and were reported previously [50]. The concentrations of EYFP, ECFP and CNA35 containing a C-terminal MESNA thioester (EYFP-MESNA, ECFP-MESNA and CNA35-MESNA) were determined by UV-vis using ε_514nm_ = 83,400 M^−1^·cm^−1^, ε_433nm_ = 32,500 M^−1^·cm^−1^ and ε_280nm_ = 33,167 M^−1^·cm^−1^, respectively. 1 L of culture resulted in yields of ~10 mg for EYFP and ECFP and ~20 mg for CNA35. Proteins were quickly frozen with liquid nitrogen and stored in the freezer until the native chemical ligation was performed.

### 4.4. Native Chemical Ligation Reactions

Native chemical ligation of Cys-Py–Im-polyamide to the thioester Nbz-peptide. Cys-Py–Im-polyamide was mixed in a slight excess (1.1 to 1 ratio) with the thioester peptide to a final concentration of 2.0 mM in a ligation buffer containing 200 mM sodium phosphate, 6 M Guanidine·HCl, 20 mM tris(2-carboxyethyl)phosphine (TCEP), 200 mM MPAA at pH 6.8 and reacted for 15 h. Excess MPAA and TCEP were removed using a solid phase extraction column (Strata-XL, Phenomenex) and the conjugate was eluted using a gradient of acetonitrile in H_2_O (both containing 0.1% TFA) and subsequently lyophilized.

Native chemical ligation of Cys-Py–Im-polyamide to ECFP-, EYFP- or CNA35-MESNA. Prior to ligation, the tertyl-butylthiol protected Cys-Py–Im-polyamide was dissolved at mM concentrations and incubated for one hour in ligation buffer containing 200 mM sodium phosphate, 180 mM TCEP, 100 mM MPAA and 2.5% DMSO at pH 6.8 to deprotect the cysteine followed by the addition of MESNA-protein resulting a 10-fold excess of Cys-Py–Im-polyamide over the MESNA-protein (3.0 mM, 0.3 mM, respectively). The reaction was performed at room temperature for 36 h. Excess Cys-Py–Im-polyamide, MPAA and TCEP were removed extensively by repeated exchanging buffer (200 mM sodium phosphate, 0.5 M NaCl at pH 6.8) using centrifugal filters (*M*_w_CO of 10 kDa).

### 4.5. Surface Plasmon Resonance

Sensor grams were obtained on a Biacore T100 (GE Healthcare, Hoevelaken, The Netherlands) using streptavidin-coated sensor chips (SA-chip, GE Healthcare). Immobilization of biotin-functionalized DNA sequences (*M*_w_G Eurofins) was achieved by the injection of 25 nM biotin-functionalized DNA at a flow rate of 2 µL·min^−1^ until the desired immobilization level of approximately 450 RUs (1 RU = 1 pg·mm^−2^) was reached. Flow channel 1 was typically chosen as the reference channel and was left unmodified. Cognate DNA sequence: CGCAT**ATGGTAT**GTGCCGCGGAAAAACCGCGGCAC**ATACCAT**AT-GCG; 3 bp mismatch: CAGTC**ATGAGCAT**GGATGCGGAAAAACCGCATCCA**TGCTCAT**-GACTG; scrambled: CGCATCTAGCACGTGCCGCGGAAAAACCGCGGCACGTGCTAGATGCG.

The maximum response of the Py–Im polyamide (conjugate) binding to the immobilized DNA can be calculated according to Equation (1) assuming a stoichiometric ratio of 1.

(1) $${RU}_{\text{max}}\;=\;\frac{M_{w}\text{Py}–\text{Im}\left(-conjugate\right)}{M_{w}DNA}\;\times RU_{immobilized}\;\times stoichiometry$$

All experiments were performed at 25 °C. Py–Im polyamide (conjugate) was dissolved in HBS-EP + 0.1% DMSO running buffer and serial dilutions were made. The Py–Im polyamide conjugates were injected from low to high concentration. Before every sample injection, a 5 min flow (25 μL·min^−1^) was performed with running buffer to obtain a stable baseline. Every sample was injected for 8 or 10 min and followed by a dissociation phase of 10 min. Regeneration of the chip was performed using 10 mM glycine pH 2.5 for 30 s. Aspecific binding and buffer effects were taken into account by subtracting the response from a reference channel not containing DNA. The collected data was analyzed using the BIAcore T100 Evaluation Software and plotted in Origin using a 1:1 binding Hill Equation (2) (*n* = 1) to obtain *K*_d_-values.

(2) $$RU\;=\;\frac{\left[\text{Py}–\text{Im}polyamide\right]}{\left[\text{Py}–\text{Im}polyamide\right]+K_{\text{d}}}\;\;\times \;{RU}_{\text{max}}$$

## Supplementary Materials

Supplementary materials can be found at http://www.mdpi.com/1422-0067/16/06/12631/s1.
