# Supplementary material for: Efficient Synthesis of Peptide and Protein Functionalized Pyrrole-Imidazole Polyamides Using Native Chemical Ligation
Source: Int J Mol Sci. 2015 Jun 4;16(6):12631–47. doi: 10.3390/ijms160612631 (PMC4490465; doi:10.3390/ijms160612631)
Supplement: Supplementary file 1 [file ijms-16-12631-s001.pdf]

Supplementary Information

Fluorescence intensity evaluation of eCFP before and after conjugation to Cys-Py-Im-polyamide.

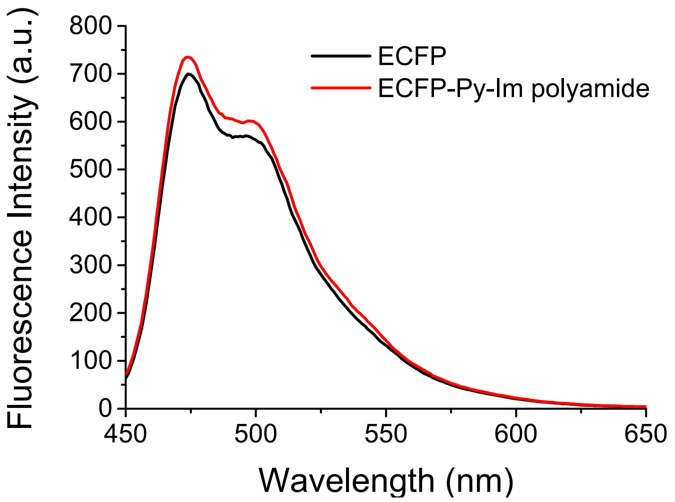

Figure S1. Fluorescence emission spectra (λ_{ex} = 420 nm) of 500 nM non-conjugated ECFP (black) and ECFP-Py-Im polyamide (red). Measurements were performed in 10 mM PBS-buffer pH 7.4 containing 1 mg·mL^{-1} BSA at room temperature. These data show that the fluorescence intensity of the fluorescent protein is not affected by the conjugation of Py-Im polyamide.
